# Academic Advising at the Faculty of Medicine, University of Gezira During 2021-2022

**DOI:** 10.7759/cureus.55267

**Published:** 2024-02-29

**Authors:** Rofayda Mansour Ahmed Mohamad, Huda Mohamed Haroun, Inshirah Mustafa Abubaker Osman

**Affiliations:** 1 Department of Preventive Medicine, King Salman Armed Forces Hospital, Tabuk, SAU; 2 Department of Pediatrics, Faculty of Medicine, University of Gezira, Wad Medani, SDN; 3 Department of Mental Health, Faculty of Medicine, University of Gezira, Wad Medani, SDN

**Keywords:** student, education, graduate, faculty, academic performance

## Abstract

Background

Since its inception, the Faculty of Medicine at Gezira University has recognized the critical role of academic advising in supporting student success. This commitment translates into a well-established advising system, fully integrated into the academic regulations and subject to continuous evaluation and improvement for maximum effectiveness and relevance. Regular orientation sessions ensure that both faculty and students are equipped to make the most of this valuable resource. However, medical students navigate a demanding path filled with unique challenges that require a robust advising program. While Gezira University has built a strong foundation, it is important to identify potential areas for further development and address any existing barriers that may prevent the system from reaching its full potential. This study was conducted to assess the academic advising program at the Faculty of Medicine, University of Gezira during the academic year 2021-2022.

Methodology

In this cross-sectional study, self-administered questionnaires were distributed among academic advisors and a sample of students at the Faculty of Medicine, University of Gezira. The advisors’ questionnaire inquired about their specialty, experience in teaching and academic advising, and other variables related to advising commitment, satisfaction, and interventions that can improve the advisors’ performance. The students’ questionnaire inquired about their batches, sex, grade point average, orientation about academic advising, communication with the advisor, satisfaction, and challenges facing the advising process.

Results

The study enrolled 70 advisors and 502 students. Most advisors were satisfied (65.7%, n = 46). The challenges facing academic advisors included the non-interest of students and inadequate training (68.6%, n = 48), lack of proper settings (65.7%, n = 46), and shortage of faculty members (60%, n = 42). About 52% (n = 261) of the students showed overall satisfaction with the advising service. The most perceived challenges by students were the difficulty of coordinating meetings (71.9%, n = 361), non-interest of the advisors (46%, n = 231), lack of benefit (16.9%, n = 85), and non-orientation of the advisors about academic rules (13.7%, n = 69).

Conclusions

The main challenges faced by academic advisors were students’ lack of interest and inadequate training, lack of appropriate settings, and lack of faculty members, while students’ perceived challenges were difficulty in coordinating meetings, advisors’ lack of interest, lack of benefit, and advisors’ lack of orientation to academic rules. The causes underlying the advisors’ and students’ dissatisfaction with academic advising should be addressed to increase their satisfaction rates. The reported barriers can be overcome by implementing an advisors’ training plan, reducing their workload, using technology, and orienting the students about the importance of academic advising and the benefits they can gain.

## Introduction

The importance of academic advising stems from the academic challenge that may face students. Sound academic advising can help students solve their problems, improve their academic performance, and achieve success [1-3]. Moreover, advising can enlighten students about their future careers. Literature on academic advising demonstrated that quality advising plays an important role in connecting students to the institution, reducing dropping out, and increasing students’ retention [4].

The academic advising system at the Faculty of Medicine, University of Gezira started with the early establishment period of the faculty and is periodically evaluated to maintain its effectiveness and usefulness. Currently, there is a software program for academic advising that is accessed by username and password for the student, the advisor, and the registration office. If the student faces any academic difficulties, the advisor report is required for registration in the forthcoming semester. Both the students and advisors are well-informed about the academic advising system. Orientation lectures are provided in the introductory course to enable the students to utilize and gain maximum benefit from the advising program. Meanwhile, an exploratory workshop is held for the advisors to maintain their understanding of the academic rules required for advising. There is an official committee working side by side with the academic advising office of the faculty, and both have an appreciated role in organizing, facilitating supporting the program, and even as mediators between the students and their advisors. The committee is represented in the faculty council with regular reports reflecting the advising scope of services.

The academic system at the Faculty of Medicine, University of Gezira, Sudan exposes students to frequent examinations, resulting in continuous stress that requires counseling and advising to manage their time. The present study aimed to evaluate the academic advising program at the Faculty of Medicine, University of Gezira during the academic year 2021-2022 in terms of perceptions and satisfaction of academic staff and students as well as challenges facing the program.

## Materials and methods

Ethical considerations

The study protocol was approved by the Ethics Committee at the Research and Medical Development Center, University of Gezira, Sudan (approval number: REC-2-2022). Participation was voluntary and informed written consent was obtained from each participant before the commencement of the study. The questionnaires were filled anonymously, and data were kept confidential.

Study design and settings

This cross-sectional study was conducted at the University of Gezira, which is considered the second governmental university in Sudan. Academic advising at the Faculty of Medicine, University of Gezira, Sudan is provided to all students.

Eligibility criteria

This study recruited students and academic advisors at the Faculty of Medicine, University of Gezira during the academic year 2021-2022. Undergraduate medical students of any age, gender, or academic year who accepted to participate were eligible to be included in the study.

Data collection tool and measured variables

The data were collected through two self-administered questionnaires with closed-ended questions. The first questionnaire was dedicated to the academic advisors at the Faculty of Medicine and included questions about the specialty, experience in teaching, and academic advising, as well as other variables related to advising commitment, willingness, satisfaction, and the interventions that can improve the advisors’ performance.

The second questionnaire assessed academic advising from the students’ viewpoint. It included questions about students’ batches, sex, grade point average, variables related to their orientation about academic advising, communication with the advisor, satisfaction with the provided advising, and the challenges facing the advising process.

To ensure the face validity of the questionnaires, revision of their content was undertaken by the academic advising committee of preventive medicine of the Saudi Board of Health Specialties, and amendments were made based on the revealed recommendations. A pilot study was conducted to ensure the content validity of the questionnaire and estimate the timing for filling it out. The pilot study included 10 academic advisors from the Saudi Board of Health Specialties. Completion of the questionnaire took about 10 to 15 minutes. The questionnaire showed good internal consistency and reliability (Cronbach’s alpha of 0.801).

Sampling technique

The academic advisor questionnaire was piloted on 10 academic advisors from the Saudi Board of Health Specialties. The total number of advisors who were targeted by the study was 70, so a comprehensive sample was taken.

Considering the second questionnaire, the sample was taken by a multistage, proportional, stratified sampling technique. At the time of questionnaire filling, there were six batches registered for the academic year 2020-2021, which were all involved in the sample. The student batches were considered as clusters (stage 1), and then stratified sampling was performed within each cluster (batch) by randomly selecting male and female students (stage 2). Care was taken to recruit the numbers of male and female participants according to the proportion of this gender within the batch.

Statistical analysis

Statistical analysis was performed using SPSS Statistics for Windows, version 26 (IBM Corp., Armonk, NY, USA). All categorical variables were summarized as frequencies and percentages. Satisfaction in the academic advisors’ group was calculated based on the answer to question 24. As for the students’ group, satisfaction was calculated from the answers to questions 10, 20, 22, and 24. The answers to each question were assigned 1-4 points for questions 10, 22, and 24 (strongly agree = 4, agree = 3, disagree = 2, strongly disagree = 1), as well as question 20 (suitable = 4, to some extent = 3, not enough = 2, no communication = 1). The points were summed up to calculate the overall satisfaction score and the median of the score was used to divide the students into two groups regarding their overall satisfaction with academic advising. The associations of satisfaction with categorical variables were assessed using Pearson’s chi-square test for independence, the Fisher-Freeman-Halton exact test, or Fisher’s exact test as appropriate. A p-value <0.05 was selected to define the significance of statistical tests.

## Results

The present study enrolled 70 academic advisors and 502 students including the six batches registered for the academic year 2020-2021 at the Faculty of Medicine, University of Gezira, Sudan.

**Figure 1:**
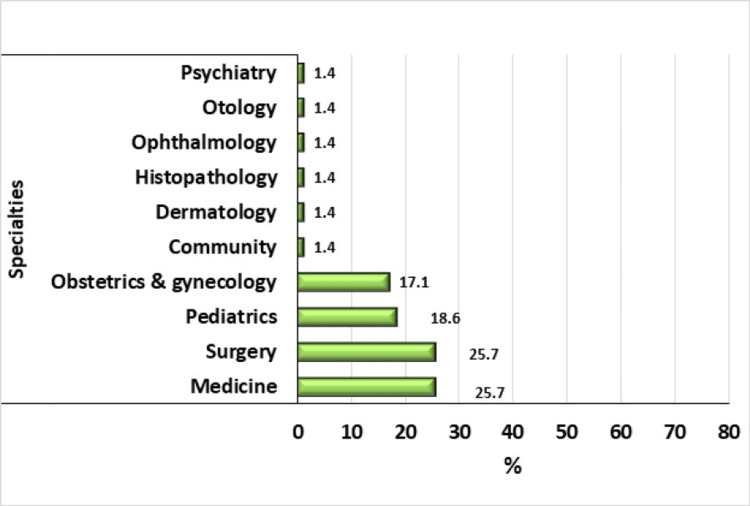
The specialties of academic advisors in the Faculty of Medicine, University of Gezira.

The most frequent specialties of the academic advisors were medicine and surgery (each 25.7% of the advisors), followed by pediatrics (18.6%) and obstetrics and gynecology (17.1%) (Figure 1).

**Figure 2:**
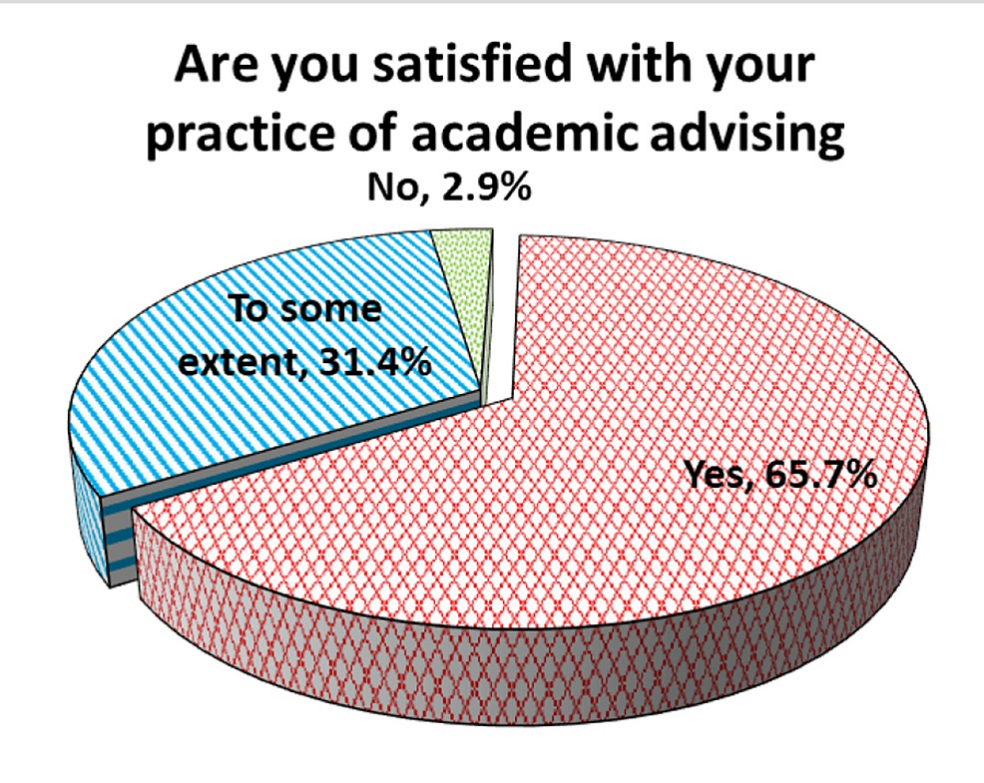
Satisfaction of the academic advisors with their practice of advising at the Faculty of Medicine, University of Gezira.

Most advisors were satisfied with their practice of academic advising (65.7%), while about one-third were satisfied to some extent and a small percentage answered negatively (2.9%) (Figure 2).

The majority of academic advisors had a doctoral degree (94.3%), were informed of their students by formal correspondence (85.7%), agreed/strongly agreed that academic advising requires training (97.1%), thought that their students need advising (94.3%), and believed that advisors’ performance can improve (82.9%). Most staff were appointed as advisors by receiving a formal letter from the administration (67.1%) and joined a training activity before working as an advisor (77.1%). About two-thirds of advisors knew satisfactorily the student-related regulations. Academic advisors were divided into two groups: those who were completely satisfied (n = 46, 65.7%) and those who were satisfied to some extent or not satisfied at all (n = 24, 34.3%). Satisfaction was significantly associated with knowledge of the regulations (p = 0.003), believing that students need more contact (p = 0.012), and the belief that the performance of advisors can improve with training (p = 0.038). In addition, a significantly higher percentage of unsatisfied academic advisors believed that their training was inadequate (p = 0.003), practiced academic advising for more than five years (p = 0.025), and knew their students only when the students themselves came to them rather than receiving a formal list from the college administration (p = 0.023) (Table 1).

**Table 1:** The association of satisfaction with the characteristics of academic advisors and their training. *: significant at p < 0.05; $+: significantly higher frequency than expected by chance; $-: significantly lower frequency than expected by chance.

Variables		Overall satisfaction of academic advisors						Statistical tests	
		Not satisfied (N = 24)		Satisfied (N = 46)		Total (N = 70)		χ^2^	P-value
		N	%	N	%	N	%		
Academic degree	Master	1	4.2	2	4.3	3	4.3	0.000	1.000
	Doctorate	22	91.7	44	95.7	66	94.3		
Academic position	Lecturer	0	0.0	3	6.5	3	4.3	1.829	0.649
	Assistant professor	10	41.7	18	39.1	28	40.0		
	Associate Professor	6	25.0	8	17.4	14	20.0		
	Professor	7	29.2	16	34.8	23	32.9		
Experience in teaching	Less than 5 years	6	25.0	12	26.1	18	25.7	3.188	0.203
	5–10 years	6	25.0	21	45.7	27	38.6		
	More than 10 years	11	45.8	13	28.3	24	34.3		
Experience in academic advising	Less than 2 years	6	25.0	12	26.1	18	25.7	7.415	0.025*
	2–5 years	4	16.7 $-	21	45.7 $+	25	35.7		
	More than 5 years	14	58.3 $+	13	28.3 $-	27	38.6		
How did you become appointed as an academic advisor?	After an orientation meeting	5	20.8	3	6.5	8	11.4	5.964	0.100
	After discussion	3	12.5	5	10.9	8	11.4		
	I received a formal letter	12	50.0	35	76.1	47	67.1		
	Others	3	12.5	2	4.3	5	7.1		
Have you joined a training activity to work as an academic advisor?	Yes	16	66.7	38	82.6	55	78.6	1.533	0.216
	No	7	29.2	8	17.4	14	20.0		
If the answer to Q7 is yes, do you believe this training was enough?	Strongly agree	2	8.3	4	8.7	6	8.6	6.614	0.028*
	Agree	11	45.8	34	73.9	45	64.3		
	Disagree	3	12.5 $+	0	0.0$-	3	4.3		
Do you think that academic advising requires training?	Strongly agree	17	70.8 $+	18	39.1 $-	35	50.0	10.551	0.003*
	Agree	5	20.8 $-	28	60.9 $+	33	47.1		
	Disagree	0	0.0	0	0.0	0	0.0		
	Strongly disagree	1	4.2	0	0.0	1	1.4		
How are you informed of the list of your students?	I participate in the students’ distribution	1	4.2	2	4.3	3	4.3	6.402	0.023*
	The list is sent to me formally	18	75.0 $-	42	91.3 $+	60	85.7		
	From the students themselves when they came to me	5	20.8 $+	1	2.2 $-	6	8.6		
Do you know the regulations and management rules that are related to students?	Yes, usually I do	10	41.7 $-	37	80.4 $+	47	67.1	11.072	0.003*
	Partially	10	41.7 $+	5	10.9 $-	15	21.4		
	No	4	16.7	4	8.7	8	11.4		
From your encounter with your groups, you think that the student’s contact with the academic advisor is	They do not seek	1	4.2	0	0.0	1	1.4	7.180	0.012*
	They do but reap no benefit	3	12.5 $+	0	0.0$-	3	4.3		
	Yes and they need more	20	83.3 $-	46	100.0 $+	66	94.3		
From your viewpoint, do you believe that academic advisors can improve their performance?	Yes	16	66.7 $-	42	91.3 $+	58	82.9	5.747	0.038*
	Some of them	6	25.0	4	8.7	10	14.3		
	No	1	4.2	0	0.0	1	1.4		

About 75% of advisors knew the number of students under their supervision. Approximately 70% knew all or most of the students personally. The number of students for each advisor varied from less than 10 in 41.4% of advisors to above 30 in 12.9%. Half of the advisors thought the students’ number was suitable; however, about one-quarter of advisors regarded the number as large. Checking the students’ results was a regular practice for 44.3% of advisors but was done occasionally by 45.7% and only in case of problems by 10%. A similar finding was observed regarding the advisors’ communication with students due to a marked change in their academic level. Providing students with feedback was done by most advisors either regularly (32.9%) or occasionally (45.7%), but 21.4% never did this. About 75% of advisors had a student with academic issues. Most advisors helped students with academic problems. Discussing future careers with students was done by 40% of advisors regularly but was rarely or never done by 48.6% and 11.4%, respectively. Support during studying or exams was provided usually upon students’ request (68.6%). Instructing about medical professionalism and ethics was a common practice for 38.6% of advisors. Half the advisors reported regular communication with students, while 44.3% were communicated by some students and 4.3% had no communication at all. The most utilized method of communication was individually (51.4%). Most students who communicated with advisors had a grade point average below 2. Satisfaction of the academic advisors was also significantly associated with knowing all the students personally (p = 0.012), advising less than 10 students (p = 0.004), and usual checking of the student’s academic results (p = 0.019), and providing feedback (p = 0.007). A significantly higher percentage of unsatisfied advisors thought they had many students in their group (p = 0.003). Satisfied advisors tended to have students with academic issues, provide help to those with academic problems, discuss future careers, help students with their studies, instruct students about medical ethics, and communicate regularly with students, particularly through face-to-face individual meetings. However, these tendencies did not show statistical significance (Table 2).

**Table 2:** The association between overall satisfaction of academic advisors and their interaction with students. *: significant at p < 0.05; GPA: grade point average; $+: significantly higher frequency than expected by chance; $-: significantly lower frequency than expected by chance.

Variables		Overall satisfaction of academic advisors						Statistical tests	
		Not satisfied (N = 24)		Satisfied (N = 46)		Total (N = 70)		χ^2^	P-value
		N	%	N	%	N	%		
Do you know the number of students under your supervision?	Yes	16	66.7	37	80.4	53	75.7	1.017	0.313
	No	7	29.2	9	19.6	16	22.9		
Do you know the students personally?	Yes, all of them	3	12.5 $-	22	47.8 $+	25	35.7	9.862	0.012*
	Yes, most of them	10	41.7	14	30.4	24	34.3		
	No, I do not know most of them	10	41.7	9	19.6	19	27.1		
	No, I do not know anyone	1	4.2	1	2.2	2	2.9		
For how many students do you provide academic advising?	Less than 10	5	20.8 $-	24	52.2 $+	29	41.4	12.613	0.004*
	10 and less than 20	5	20.8	12	26.1	17	24.3		
	20 and less than 30	9	37.5 $+	3	6.5 $-	12	17.1		
	More than 30	4	16.7	5	10.9	9	12.9		
What is your opinion about this number of students?	Very few	1	4.2	1	2.2	2	2.9	14.181	0.003*
	Few	1	4.2	10	21.7	11	15.7		
	Suitable	10	41.7	29	63.0	39	55.7		
	Lots of	8	33.3 $+	4	8.7 $-	12	17.1		
	Huge	4	16.7 $+	1	2.2 $-	5	7.1		
Do you check the results of students in your group?	Yes usually	6	25.0 $-	25	54.3 $+	31	44.3	7.672	0.019*
	Sometimes	13	54.2	19	41.3	32	45.7		
	Only when they have an academic problem	5	20.8 $+	2	4.3 $-	7	10.0		
	No	0	0.0	0	0.0	0	0.0		
Have you ever communicated with a student due to a marked change in the academic level?	Yes, usually I do	8	33.3	20	43.5	28	40.0	1.913	0.392
	Few times	13	54.2	24	52.2	37	52.9		
	No, I have no idea about their performance	3	12.5	2	4.3	5	7.1		
Do you provide feedback to your students about their results or performance in academic activities?	Yes	2	8.3 $-	21	45.7 $+	23	32.9	9.956	0.007*
	Sometimes	15	62.5	17	37.0	32	45.7		
	No	7	29.2	8	17.4	15	21.4		
Do you have a student with academic issues?	Yes	16	66.7	36	78.3	52	74.3	2.027	0.320
	No	6	25.0	9	19.6	15	21.4		
	I do not know	2	8.3	1	2.2	3	4.3		
Have you ever helped a student with an academic problem?	Yes	21	87.5	45	97.8	66	94.3	FE	0.113
	No	3	12.5	1	2.2	4	5.7		
Do you discuss future careers with your students?	Yes, usually I do	8	33.3	20	43.5	28	40.0	1.378	0.523
	Rarely	12	50.0	22	47.8	34	48.6		
	No	4	16.7	4	8.7	8	11.4		
Do you help students in studying or before the exams?	Yes, usually I do	6	25.0	13	28.3	19	27.1	1.540	0.544
	If they ask	16	66.7	32	69.6	48	68.6		
	No	2	8.3	1	2.2	3	4.3		
Do you instruct them about the medical profession and ethics?	Yes, usually I do	7	29.2	20	43.5	27	38.6	6.285	0.077
	Yes, sometimes	11	45.8	8	17.4	19	27.1		
	If they ask	6	25.0	16	34.8	22	31.4		
	No	0	0.0	2	4.3	2	2.9		
Do students communicate regularly with you?	Yes	9	37.5	27	58.7	36	51.4	3.131	0.193
	Some of them	14	58.3	17	37.0	31	44.3		
	No	1	4.2	2	4.3	3	4.3		
If the answer to Q 26 is yes, how do they communicate?	Individually	10	41.7	26	56.5	36	51.4	3.218	0.366
	As group	5	20.8	8	17.4	13	18.6		
	Social media	4	16.7	4	8.7	8	11.4		
	More than one method	3	12.5	2	4.3	5	7.2		
From your group, which students are more communicative (regarding their GPA)?	Less than 2	16	66.7	29	63.0	45	64.3	0.545	0.808
	2–3	5	20.8	10	21.7	15	21.4		
	More than 3	2	8.3	7	15.2	9	12.9		

**Figure 3:**
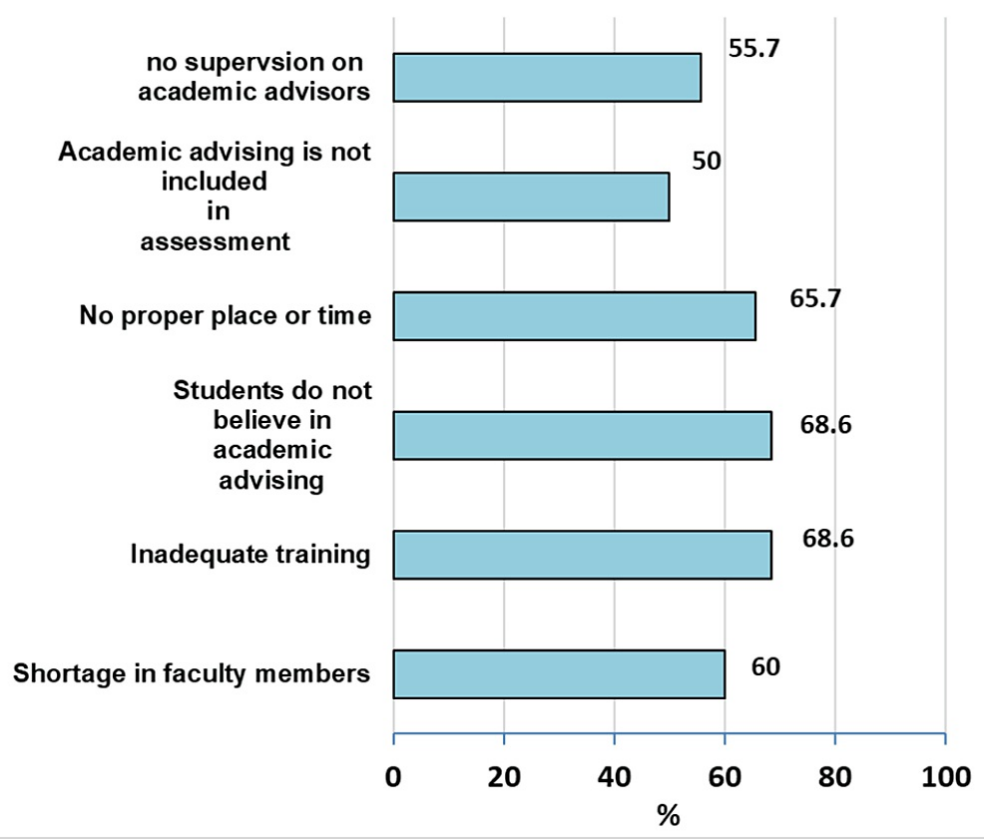
Challenges facing academic advising at the Faculty of Medicine, University of Gezira: academic advisors’ perspectives.

According to the academic advisors, challenges to academic advising included the non-belief of students in advising and inadequate training of advisors (68.6% each), lack of proper place or time (65.7%), shortage of faculty members (60%), lack of supervision on academic advisors (55.7%), and the non-inclusion of academic advising in students’ assessment (50%) (Figure 3).

Regarding medical students, most students strongly agreed/agreed that their academic advisor is doing his job (59.2%). However, 42.8% regarded their communication with the advisor as being not enough, and 30.7% reported a lack of communication. Nearly half the students (54%) agreed/strongly agreed that academic advising is beneficial to them, but about one-third (34.2%) were willing to change their academic advisor if they had the chance. The students’ overall satisfaction scores ranged from 4 to 15, with a median of 10. Students were divided into two groups: satisfied (score ≥ 10, n = 261, 52.0%) and unsatisfied (score > 10, n = 217, 43.2%) (Table 3).

**Table 3:** Satisfaction of students with academic advising at the Faculty of Medicine, University of Gezira.

Variables		N	%
Do you believe that your academic advisor is doing his job?	Strongly agree	44	8.8
	Agree	253	50.4
	Disagree	146	29.1
	Strongly disagree	58	11.6
	No response	1	0.2
How do you evaluate your communication with him?	Suitable	36	7.2
	To some extent	93	18.5
	No, not enough	215	42.8
	No communication	154	30.7
	No response	4	0.8
Do you believe that academic advising is beneficial for you?	Strongly agree	62	12.4
	Agree	209	41.6
	Disagree	115	22.9
	Strongly disagree	106	21.1
	No response	10	2.0
If you find a chance to change your academic advisor, will you do it?	Strongly agree	96	19.1
	Agree	76	15.1
	Disagree	233	46.4
	Strongly disagree	81	16.1
	No response	16	3.2
Satisfaction	Unsatisfied	217	43.2
	Satisfied	261	52.0

**Figure 4:**
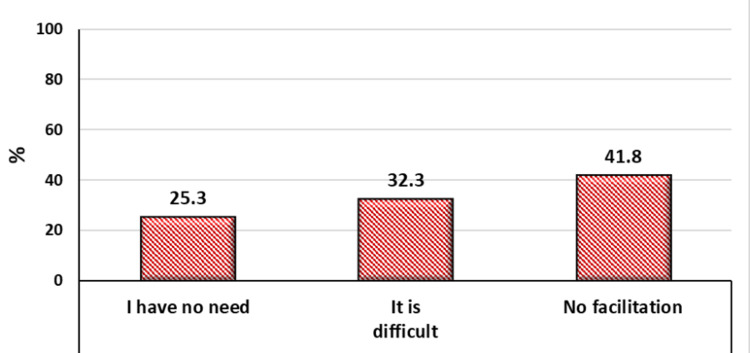
Reasons for poor communication of students with their academic advisors.

Most students knew their academic advisors (94.4%) and agreed/strongly agreed that academic advising is important (93.8%). About 44% of students were in contact with their advisors, while one-third rarely communicated, and one-fifth never did this. The most common reasons for poor communication were the lack of facilitation (41.8%), difficulty (32.3%), and the lack of need (25.3%) (Figure 4).

The most common methods of communication were individual meetings (51.4%) and social media (43.4%). Most students informed their advisors of their exam results, either regularly (33.1%) or sometimes (40.4%), whereas one quarter never did so. However, only half of the students reported getting a benefit by informing advisors of exam results, while about 31% perceived no benefits. Students’ satisfaction was significantly associated with believing in the importance of academic advising (p < 0.001), communication with the academic advisor (p < 0.001), communication through social media (p = 0.006), informing the advisor of their results and perceiving benefits (p < 0.001) (Table 4).

**Table 4:** Association of students’ satisfaction with their characteristics and their communication with their advisors. *: significant at p < 0.05; GPA: grade point average; $+: significantly higher frequency than expected by chance; $-: significantly lower frequency than expected by chance.

Variables		Overall satisfaction				All students		Statistical tests	
		Unsatisfied (score <10)		Satisfied (score ≥10)				χ^2^	P-value
		N	%	N	%	N	%		
Batch	38	37	17.1	45	17.2	87	17.3	4.995	0.417
	39	38	17.5	55	21.1	93	18.5		
	40	38	17.5	38	14.6	81	16.1		
	41	47	21.7	41	15.7	91	18.1		
	42	29	13.4	45	17.2	82	16.3		
	43	28	12.9	37	14.2	68	13.5		
Sex	Female	124	57.1	159	60.9	295	58.8	0.700	0.403
	Male	93	42.9	102	39.1	207	41.2		
GPA	Less than 2	12	5.5	7	2.7	20	4.0	2.760	0.252
	2–3	65	30.0	72	27.6	147	29.3		
	More than 3	138	63.6	174	66.7	325	64.7		
Do you know your academic advisor?	Yes	194	89.4 $-	257	98.5 $+	474	94.4	18.275	<0.001*
	No	23	10.6 $+	4	1.5 $-	28	5.6		
Do you believe that academic advising is important?	Strongly agree	94	43.3	117	44.8	223	44.4	32.781	<0.001*
	Agree	96	44.2	140	53.6	248	49.4		
	Disagree	25	11.5 $+	1	0.4 $-	26	5.2		
	Strongly disagree	2	0.9	3	1.1	5	1.0		
Do you communicate with your academic advisor?	Yes, usually	8	3.7 $-	33	12.6 $+	42	8.4	73.221	<0.001*
	Yes	64	29.5 $-	105	40.2 $+	178	35.5		
	Yes, but rarely	63	29.0 $-	105	40.2 $+	176	35.1		
	No	82	37.8 $+	18	6.9 $-	106	21.1		
How do you communicate with him?	Individually	123	56.7 $+	124	47.5 $-	258	51.4	5.440	0.020*
	As a group	19	8.8	29	11.1	48	9.6	0.567	0.452
	Social media	77	35.5 $-	128	49.0 $+	218	43.4	7.479	0.006*
Do you inform him of your results?	Yes, usually	64	29.5	94	36.0	166	33.1	63.278	<0.001*
	Sometimes	58	26.7 $-	136	52.1 $+	203	40.4		
	No	92	42.4 $+	31	11.9 $-	129	25.7		
If you answered yes in Q11, does he benefit you?	Strongly agree	4	3.4 $-	38	16.7 $+	43	11.8	100.166	<0.001*
	Agree	38	31.9 $-	157	68.9 $+	205	56.3		
	Disagree	53	44.5 $+	31	13.6 $-	90	24.7		
	Strongly disagree	24	20.2 $+	2	0.9 $-	26	7.1		

Most students never discussed their future careers with academic advisors (83.5%) and 5.8% found the discussion useless. About 42% of students experienced an academic problem and needed their advisors’ help. Among those with problems, 54.6% sought the advisors’ help, 19.5% tried to reach the advisor without success, and 10.2% received no help after contacting the advisor. Only 21% sought advisors’ help in the learning process. Most students stated that they did not receive help from their advisors concerning the academic rules or in making decisions (61.6%). Likewise, 68.9% of students never consulted their advisors on academic issues. About 78% agreed/strongly agreed that they need more communication with the advisors. Students’ satisfaction was significantly associated with discussing their future careers with their advisors (p < 0.001), needing advice on an academic problem (p < 0.001), seeking help during the learning process (p < 0.001), receiving help in exploring academic rules (p < 0.001), and consulting regarding an academic issue (p < 0.001). A significantly higher percentage of satisfied students agreed that they needed more communication with their advisors (p = 0.002) (Table 5).

**Table 5:** Association of students’ satisfaction with the received advising from their advisors. *: significant at p < 0.05; $+: significantly higher frequency than expected by chance; $-: significantly lower frequency than expected by chance.

Variables		Overall satisfaction				All students		Statistical tests	
		Unsatisfied (score <10)		Satisfied (score ≥ 10)				χ^2^	P-value
		N	%	N	%	N	%		
Do you discuss with him your future career?	Yes, usually	4	1.8$-	43	16.5 $+	50	10.0	31.602	<0.001*
	Yes, but it is not useful	9	4.1	19	7.3	29	5.8		
	No	201	92.6 $+	199	76.2 $-	419	83.5		
Have you ever experienced an academic problem and needed your advisor?	Yes	69	31.8 $-	126	48.3 $+	210	41.8	13.043	<0.001*
	No	147	67.7 $+	135	51.7 $-	291	58.0		
If your answer is yes to the previous question, do you seek his help?	Yes	17	24.6 $-	91	74.6 $+	112	54.6	48.041	<0.001*
	I tried but could not	20	29.0	18	14.8	40	19.5		
	I contacted him but he did not help me	15	21.7 $+	5	4.1 $-	21	10.2		
	No, I did not contact him	17	24.6 $+	8	6.6 $-	32	15. 6		
Do you seek his help in the learning process for example asking him about references?	Yes Strongly	0	0.0$-	16	6.1 $+	16	3.2	78.216	<0.001*
	Yes	15	6.9 $-	71	27.2 $+	90	17.9		
	No	123	56.7	147	56.3	283	56.4		
	Strongly no	76	35.0 $+	24	9.2 $-	104	20.7		
Has he ever helped you in exploring academic rules or helped you in making a decision?	Yes, usually	2	0.9$-	25	9.6 $+	28	5.6	102.371	<0.001*
	Sometimes	27	12.4 $-	125	47.9 $+	159	31.7		
	No	188	86.6 $+	107	41.0 $-	309	61.6		
Have you ever consulted him on an academic issue?	Yes Strongly	1	0.5$-	16	6.1 $+	17	3.4	102.130	<0.001*
	Yes	29	13.4 $-	100	38.3 $+	134	26.7		
	No	110	50.7	134	51.3	256	51.0		
	Strongly no	76	35.0 $+	10	3.8 $-	90	17.9		
Do you need more communication with him?	Strongly agree	74	34.1 $+	66	25.3 $-	146	29.1	14.841	0.002*
	Agree	91	41.9 $-	144	55.2 $+	246	49.0		
	Disagree	33	15.2	46	17.6	82	16.3		
	Strongly disagree	15	6.9 $+	5	1.9 $-	20	4.0		

**Figure 5:**
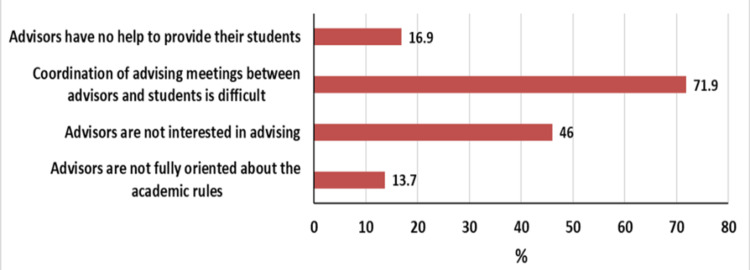
Challenges facing academic advising at the Faculty of Medicine, University of Gezira: students’ perspectives.

According to medical students, the most frequent challenges to academic advising were the difficulty of coordinating meetings between the advisor and students (71.9%), lack of interest of the advisors (46%), that advisors have no help to provide (16.9%), and non-orientation of the advisors about the academic rules (13.7%) (Figure 5).

## Discussion

This study aimed to evaluate the academic advising program at the Faculty of Medicine, Gezira University during the 2021-2022 academic year.

Our results showed that most advisors (94.3%) believed that their students need advising and that academic advising requires training (97.1%). In addition, 82.9% believed that advisors’ performance could improve. However, only 77.1% of advisors have received training before working. For academic advisors to perform their responsibilities, they should perceive the importance of advising as an integral part of the institution’s mission. Such an attitude enables advisors to tackle their duties and dedicate proper time to advising as they do for other work duties.

Training of the advisors should provide a theoretical basis for the different approaches of advising (e.g., the prescriptive and developmental methods) and the way of using different approaches to ensure fulfilling the students’ expectations. Training can also include using technological methods to facilitate meetings with students or to arrange their schedules [5]. Online support tools can enable the advisor to solve the students’ issues related to courses [6]. Lai-Yeung [7] suggested that the training program should include a list of essential elements, such as communication skills, time management, and knowledge about administrative issues.

From the student’s perspective, most students (93.8%) perceived the importance of academic advising, but only 54% regarded it as being beneficial. Moreover, 30% of the students who experienced an academic problem either failed to reach their advisors or received no help after communication. The responses of the advisors and the students to the questionnaires suggest that academic advising is not fully utilized by students either due to factors related to the advisors (e.g., defective knowledge about regulations, difficulty to contact), students (e.g., not informing the advisors with their academic issues), or the settings (e.g., improper time or place for meetings).

An important aspect of academic advising is possessing a thorough knowledge of student-related regulations so that the advisors can help students decide on their courses and schedules besides helping them to solve the academic issues they may face [6]. Nevertheless, only two-thirds of the advisors knew satisfactorily student-related regulations. This defect was mirrored by the students’ questionnaire where 61.6% of students stated that they did not receive help from their advisors concerning the academic rules or in making decisions. Further, 13.7% of students listed this as one of the challenges facing academic advising. This lack of administrative knowledge was stated by advisors in a previous study by Karr-Lilienthal et al. [8].

Most academic advisors at the Faculty of Medicine, University of Gezira performed several tasks related to advising, including checking the students’ results, contacting students with lowered academic performance, providing feedback about their achievements, and helping those with academic issues. However, only 30-40% of advisors perform these tasks regularly, while the remaining advisors do so occasionally or never. The academic advising system should ensure that all advisors maintain regular contact with their students and perform their tasks. Advisors should also discuss future careers and provide instruction on medical professionalism and ethics. It is important to avoid overlooking these aspects of advising.

Regarding the communication between advisors and students, half of the advisors reported regular communication with students, while 44.3% were communicated by some students and 4.3% had no communication at all. This finding agrees with students’ self-reported contact with advisors, with 44% regularly communicating with them, one-third rarely communicating, and one-fifth never doing this. The most common reasons for poor communication were the lack of facilitations (41.8%), difficulty in arranging a meeting (32.3%), and the perceived lack of need (25.3%).

The results of the present study also indicate the underutilization of the academic advising service by the students. Only 21% sought the advisors’ help in the learning process while 68.9% never consulted their advisors on academic issues. Other studies have reported that students tend to receive help in the learning process from their colleagues rather than their advisors. Burk and Bender [9] in the United States found that 39% of students depended on advisors for support compared to 87% depending on classmates. Al-Ansari et al. [10] in Saudi Arabia reported that most students sought advice on academic issues from their colleagues, and only 7.6% depended on academic advisors.

Most students either regarded their communication with the advisor as not enough (42.8%) or reported a lack of communication (30.7%). This can be explained by the shortage of academic advisors relative to the number of students. It also indicates the student’s need for more communication with their advisors (as expressed by 78% of students), and their wish to increase the frequency of meetings or to facilitate contact with advisors if they encounter an academic problem. Most students who communicated with advisors had a grade point average below 2. This may reflect their need for academic support to overcome the difficulties they experience in medical education and training. It could also reflect a flawed perception of academic advising as being related only to academic support.

The most utilized method of communication was individually (51.4%). This percentage was slightly lower than that reported by Issrani et al. [11] at Jouf University, Saudi Arabia (84.4%). This could be explained in light of the considerable number of students contacting their advisors through social media (43.4%). It seems that social media offered a convenient means of communication for these students and overcame the difficulties of arranging a place and time for a person-to-person meeting [12]. Previous studies [6,13-15] showed that students regard online communication as beneficial for contacting their advisors. Further research is required to evaluate the satisfaction and effectiveness of social media as a means of communication with advisors at our institution compared to face-to-face meetings.

There is a paucity of literature regarding the satisfaction of academic advisors with advising tasks. Most studies explored students’ satisfaction only. Most advisors in this study were satisfied (65.7%), while about one-third were satisfied to some extent, and 2.9% were dissatisfied. This finding agrees with Donnelly [16] who reported that most academic advisors were satisfied with their jobs (79%), 8% were dissatisfied, and 13% were neutral.

Satisfaction with academic advising was significantly associated with knowledge of the regulations, believing that students need more contact, believing that training can improve the advisors’ performance, knowing all the students personally, advising fewer than 10 students, and usual checking of the student’s academic results, and providing feedback. A significantly higher percentage of unsatisfied advisors thought they had a large number of students in their group, believed that their training was inadequate, and practiced academic advising for more than five years. The results suggest that advisors who spent a long duration performing academic advising become eventually unsatisfied. This can be related to the increased work burden for the senior faculty members which may make them unable to manage their time in the face of the several work tasks. These findings can help design an improvement plan for the academic advising program by reducing the number of students assigned to an advisor to facilitate personal communications and establish a strong advisor-student bond. Increasing the frequency of meetings is recommended as most students reported the need for more communication. A higher frequency of communication can strengthen the student-advisor relationship [17,18].

Regarding students’ overall satisfaction with academic advising, 52.0% were satisfied and 43.2% were unsatisfied. Moreover, 34.2% of students were willing to change their academic advisor if they had the chance. Previous studies reported varying rates of student satisfaction, presumably due to differences in the tools used to assess satisfaction besides variations in culture and the demographics of students. Allard and Parashar [19] reported that 61% of students in the Northeast United States were satisfied. Al-Asmi & Thumiki [20] at an institution in Muscat, Oman reported that 39.3% of students were satisfied and 44% were dissatisfied. Al-Ansari et al. [10] in Saudi Arabia reported a very low satisfaction rate (17.2%) whereas 47.7% of students were very or somewhat dissatisfied. Chemishanova [21] in the United States reported that dissatisfied and satisfied students accounted for 20.8% and 58.4%, respectively. Issrani et al. [11] found that about 42% of students were satisfied with the academic advising they received, while 27.7% were neutral and 30% were dissatisfied.

Students’ satisfaction in the present study was significantly associated with believing in the importance of academic advising, communication through social media, informing the advisor of their results and perceiving benefits, discussing their future career with their advisors, needing advice on an academic problem, seeking help during the learning process, receiving help in exploring academic rules, and consulting regarding an academic issue. These findings highlight the aspects of academic advising that are important to the students and that contribute to their satisfaction. These aspects should receive their due importance in the training of academic advisors. Students may become more regular in communication and perceive more benefits if these aspects are fulfilled in their meetings with their advisors.

Satisfaction was not associated with students’ gender in the current study. However, contradictory results were reported by Issrani et al. [11] who reported a higher satisfaction among female students. Meanwhile, Afshar and Dhiman [22] reported that female students rated academic advising lower than did the male cohorts. Satisfaction was not associated with the academic level in our sample of students. On the other hand, other studies found that the new students were more positive about academic advising than seniors [11,22].

This study explored the challenges facing academic advising. The challenges perceived by academic advisors included the non-belief of students in advising and inadequate training of advisors (68.6% each), lack of proper place or time (65.7%), shortage of faculty members (60%), lack of supervision of academic advisors (55.7%), and the non-inclusion of academic advising in students’ assessment (50%). The challenges selected by medical students were the difficulty of coordinating meetings between the advisor and students (71.9%), lack of interest of the advisors (46%), that advisors have no help to provide (16.9%), and non-orientation of the advisors about the academic rules (13.7%). As stated earlier, an improvement plan or even a plan for a new advising program should tackle these challenges to maximize the benefit and satisfaction of the students as well as the advising staff.

Cotten and Wilson [23] reported that the barriers perceived by students were limited time, uncertainty about the advisor’s interest in meeting, insecurity, and students’ unawareness of the topics an advisor can discuss with them beyond course needs. Issrani et al. [11] stated that the student-reported weaknesses of academic advising included the inability of the advisors to arrange an appropriate time for the meeting, lack of desire and interest by advisors, defective up-to-date information, unavailability of advisors due to their other responsibilities, and inability to communicate effectively with students.

Limitations

The results of the present study should be interpreted with caution due to the encountered limitations. The cross-sectional design of the study prevented the establishment of causality between staff or student satisfaction and potential causes. Additionally, the sample size of academic advisors was relatively small as the study was conducted in a single institution. Therefore, we recommend that future studies include multiple institutions. Furthermore, it may be preferable to use a satisfaction score based on multiple questions to assess the satisfaction of academic advisors, rather than relying on a single question.

## Conclusions

The rate of advisors’ satisfaction was slightly higher than that of the students, calling for measures to increase the satisfaction of both parties. The causes underlying the advisors’ and students’ dissatisfaction with academic advising should be addressed. The reported barriers can be overcome by implementing an advisors’ training plan, reducing their workloads, using technology, and orienting the students with the importance of academic advising and the benefits they can gain.
